# The uPA/uPAR System Orchestrates the Inflammatory Response, Vascular Homeostasis, and Immune System in Fibrosis Progression

**DOI:** 10.3390/ijms24021796

**Published:** 2023-01-16

**Authors:** Yosuke Kanno

**Affiliations:** Department of Clinical Pathological Biochemistry, Faculty of Pharmaceutical Science, Doshisha Women’s College of Liberal Arts, Kyoto 610-0395, Japan; ykanno@dwc.doshisha.ac.jp; Tel.: +81-0774-65-8629

**Keywords:** uPA, uPAR, plasmin, fibrosis 23

## Abstract

Fibrotic diseases, such as systemic sclerosis (SSc), idiopathic pulmonary fibrosis, renal fibrosis and liver cirrhosis are characterized by tissue overgrowth due to excessive extracellular matrix (ECM) deposition. Fibrosis progression is caused by ECM overproduction and the inhibition of ECM degradation due to several events, including inflammation, vascular endothelial dysfunction, and immune abnormalities. Recently, it has been reported that urokinase plasminogen activator (uPA) and its receptor (uPAR), known to be fibrinolytic factors, orchestrate the inflammatory response, vascular homeostasis, and immune homeostasis system. The uPA/uPAR system may show promise as a potential therapeutic target for fibrotic diseases. This review considers the role of the uPA/uPAR system in the progression of fibrotic diseases.

## 1. Introduction

**Figure 1:**
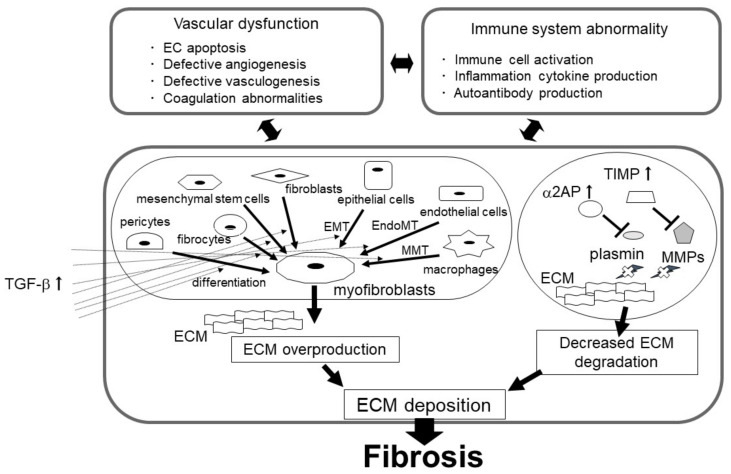
The mechanism of fibrosis progression.

Fibrosis is characterized by the deposition of excessive extracellular matrix (ECM) components, such as collagen and fibronectin. Fibrosis is a prominent pathological feature of chronic autoimmune diseases (systemic sclerosis [SSc], systemic lupus erythematosus [SLE], rheumatoid arthritis, ulcerative colitis, Crohn’s disease, myelofibrosis) and chronic kidney disease (CKD; diabetes mellitus, hypertension, infection, glomerulonephritis), and causes organ dysfunction or death [1,2]. The common feature of all fibrotic diseases is the deposition and activation of myofibroblasts, which causes excessive ECM production [3]. Myofibroblast deposition is induced by the differentiation of tissue-resident fibroblast and bone marrow-derived mesenchymal stem cells (MSCs), epithelial-to-mesenchymal transition (EMT), endothelial-to-mesenchymal transition (EndoMT), and macrophage-to-myofibroblast transition (MMT) [4,5,6,7,8,9]. In contrast, matrix metalloproteinases (MMPs) and plasmin contribute to ECM degradation [10,11], and MMPs are also associated with fibrosis progression [12,13,14,15,16,17]. Tissue inhibitor of MMPs (TIMP) and plasmin inhibitor (α2-antiplasmin [α2AP]) expression is elevated in fibrotic tissues [18,19,20,21,22,23]. Inhibitors of MMPs, plasmin, and urokinase plasminogen activator (uPA) (TIMP, α2AP, and plasminogen activator inhibitor-1 [PAI-1], respectively) cause impaired ECM degradation. Together, ECM overproduction and its impaired degradation induce fibrosis progression. Vascular dysfunction (endothelial cell [EC] damage, defective angiogenesis, and coagulation abnormalities) and abnormalities in the immune system (immune activation, T-cells, B-cells, macrophages infiltration, and autoantibodies production) affect ECM deposition and fibrosis progression (Figure 1) [1,11,24].

**Figure 2:**
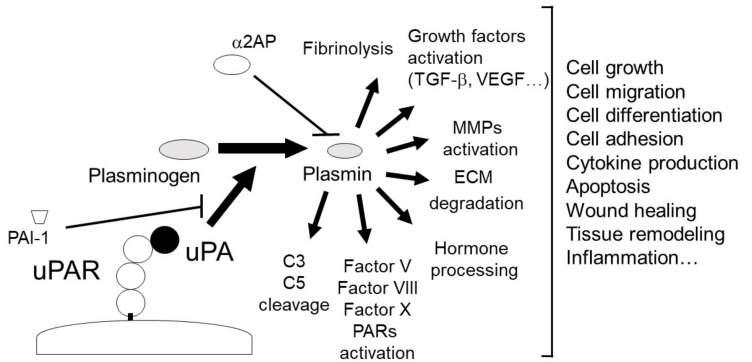
The functions of plasmin.

uPA and its receptor (uPAR) convert plasminogen (Plg) into plasmin [25]. Plasmin regulates fibrinolysis, the activation of growth factors, activation of MMPs, ECM degradation, hormone processing, and activation of factor V, factor VIII, factor X, and protease-activated receptors (PARs). Plasmin is associated with multiple cell functions, cytokine production, apoptosis, and tissue remodeling, as well as inflammation through various mechanisms (Figure 2) [11,26,27,28,29,30,31]. In contrast, uPA and uPAR interact with transmembrane proteins, such as integrins, and regulate cell growth, migration, differentiation, and adhesion [32,33,34,35,36]. uPA and uPAR mediate inflammation and the immune response, tissue remodeling, and angiogenesis, and are involved in the progression of fibrotic diseases, such as SSc and rheumatoid arthritis [32,34,37,38,39]. This review describes the roles of uPA and uPAR in the pathogenesis of fibrotic diseases.

## 2. The uPA and uPAR System

The uPA and uPAR system can convert Plg into plasmin or activate intercellular signaling and regulates fibrinolysis, cell proliferation, migration, differentiation, and adhesion. The uPA/uPAR system is associated with the immune response, angiogenesis, inflammation, tissue remodeling, bone metabolism, glucose metabolism, and fibrosis progression [13,36,37,40,41,42,43,44].

### 2.1. uPA

uPA is a single-chain serine protease that can cleave and activate Plg into plasmin by binding to uPAR [45]. uPA is secreted as a single-chain glycosylated zymogen called pro-uPA, and pro-uPA is activated by several proteinases, such as kallikrein, stromelysin, and plasmin [45]. uPA consists of three domains: an N-terminal epidermal growth factor (EGF)-like domain (which together with the N-terminal domain form the amino-terminal fragment [ATF]), and catalytic serine protease domain [46,47]. uPA is expressed in many types of cells, including leukocytes, macrophages, tumor cells, and fibroblasts [40]. uPA can bind to the uPAR D1 domain through its EGF-like domain [47]. The uPA/uPAR interaction mediates various signal pathways, such as phosphatidylinositol 3-kinase (PI3K)/Akt, AMP-activated protein kinase (AMPK), extracellular-signal-regulated kinase (ERK), and c-Jun N-terminal kinase (JNK) [27,34,48]. PAI-1 blocks uPA and inhibits plasmin production and the fibrinolytic system [49].

### 2.2. uPAR

**Figure 3:**
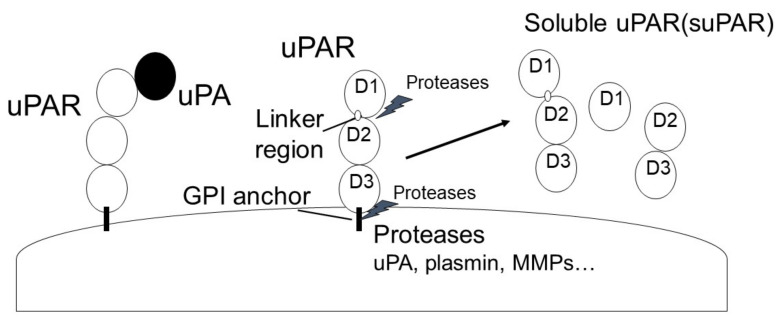
The structure of uPAR.

**Figure 4:**
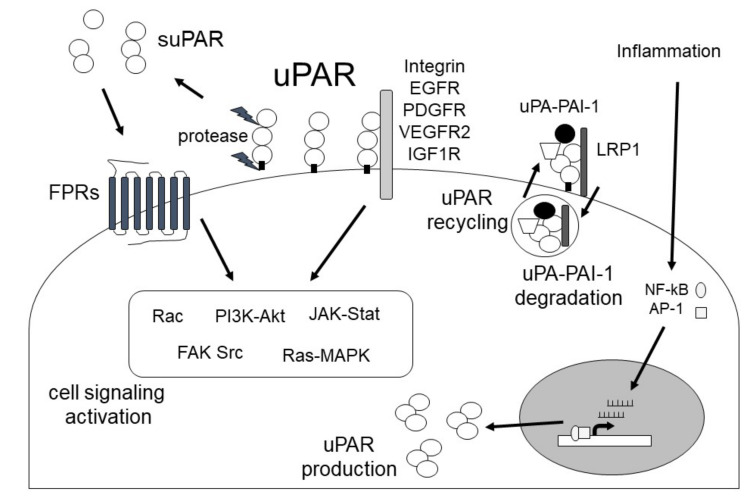
The multiple functions of uPAR.

**Figure 5:**
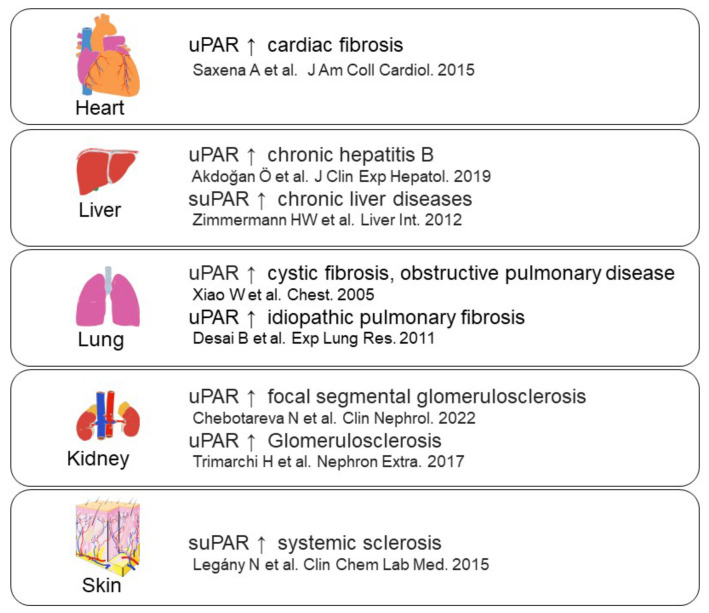
uPAR expression in fibrosis-related diseases [56,57,58,59,60,61,62,63].

uPAR is a glycosyl-phosphatidyl-inositol anchored (GPI) membrane protein that consists of three domains: D1 (residues 1–92), D2 (residues 93–191) and D3 (residues 192–283) [50]. uPAR is cleaved between the D1 and D2 domains (linker region) and the GPI-anchor domain by several proteases, such as uPA, plasmin, MMPs, and GPI-specific phospholipase D, and then forms soluble uPAR (suPAR; full length D1-D3, D2D3, and D1) (Figure 3) [51]. uPAR can interact with various transmembrane receptors, including integrins, epidermal growth factor receptor (EGFR), platelet-derived growth factor receptors (PDGFR), vascular endothelial growth factor receptor 2 (VEGFR2), and insulin-like growth factor 1 receptor (IGF1R), as well as regulate signal transduction [32,50]. In addition, uPAR can interact with low-density lipoprotein receptor (LRP), and this uPAR/LRP interaction is associated with uPAR recycling [52]. uPAR can also bind to vitronectin, and the vitronectin/uPA/suPAR complex leads to increased PA activity [53]. Furthermore, suPAR can activate formyl peptide receptor (FPR) [54]. The functions of uPAR regulate various signaling pathways, such as PI3K/Akt, focal adhesion kinase (FAK), and Janus kinase (JAK)-signal transducer and activator of transcription protein (STAT), and is associated with the immune response, angiogenesis, inflammation, and fibrosis (Figure 4). uPAR is expressed on a variety of cells, including fibroblasts, monocytes, macrophages, keratinocytes, neurons, ECs and smooth muscle cells [27], and uPAR expression is induced by inflammation [33,55]. It has been reported that increased uPAR expression is observed in many fibrosis-related diseases, including cardiac fibrosis, idiopathic pulmonary fibrosis, and SSc (Figure 5) [56,57,58,59,60,61,62,63].

## 3. The Role of the uPA/uPAR System in Fibrosis

Fibrosis is characterized by ECM deposition due to the overproduction of ECM and the inhibition of ECM degradation. Many fibrotic events, such as vascular endothelial dysfunction and immune abnormalities, are associated with the activation and differentiation of myofibroblasts and the inhibition of ECM-depredating proteases, including MMPs and plasmin, which cause ECM deposition [9,11,64,65,66,67].

Induction of uPA activity attenuates pulmonary fibrosis in fibrosis model mice [67]. In addition, transplantation of uPA gene attenuates liver fibrosis in liver fibrosis model rats [68]. uPAR deficiency induces perivascular fibrosis, dermal fibrosis, and pulmonary fibrosis in mice [13,69,70] and accelerates renal fibrosis in obstructive nephropathy model mice [71]. In contrast, suPAR is elevated in focal segmental glomerulosclerosis (FSGS) [72], and uPAR deficiency attenuates LPS-induced glomerulosclerosis [73]. uPAR isoform 2 transgenic mice exhibit glomerulosclerosis and kidney dysfunction [74]. In addition, UPARANT (Ac-L-Arg-Aib-L-Arg-D-Cα(Me)Phe-NH_2_), which blocks uPAR binding to the FPR, restores STZ-induced renal fibrosis [75]; therefore, uPAR affects renal fibrosis progression.

Plasmin not only degrades fibrin but also induces MMP and PAR activity. Fibrin and PAR activation promotes fibrosis, and MMPs play an important role in ECM degradation [76,77]. In addition, plasmin-induced hepatocyte growth factor (HGF) activation and vascular endothelial growth factor (VEGF) release may affect fibrosis progression [78,79]. Furthermore, α2AP and PAI-1 deficiency attenuated fibrosis progression in fibrosis model mice [21,78,80,81,82]. These data suggest that the uPA/uPAR system plays a pivotal role in the progression of fibrosis through multiple plasmin-dependent and plasmin-independent mechanisms.

### 3.1. Myofibroblasts in Fibrosis

Myofibroblasts contribute to excessive ECM production and play a pivotal role in fibrotic disorders [3,66]. It has been reported that various cell types, including fibroblasts, pericytes, bone-marrow-derived fibrocytes, tissue-derived MSCs, ECs, epithelial cells, and macrophages give rise to myofibroblasts [83]. Inflammation or mechanical stresses induce pro-fibrotic factors, including transforming growth factor-β (TGF-β), platelet-derived growth factor (PDGF), and interleukins (ILs). Myofibroblasts are raised from MSCs, pericytes and pre-adipocytes, epithelial cells, ECs, and macrophages through differentiation, EMT, EndoMT, or MMT induced by pro-fibrotic factors [3,5,84,85,86,87,88,89]. Furthermore, the apoptosis resistance of myofibroblasts has been observed in several fibrotic tissues [66]. TGF-β and PDGF inhibit myofibroblast apoptosis [66,90,91]. In contrast, HGF induces myofibroblast apoptosis through FAK-ERK signaling, and suppresses fibrosis progression [92,93]. The evasion of apoptosis in myofibroblasts may play a pivotal role in the progression of fibrosis.

#### 3.1.1. The uPA/uPAR System and Myofibroblasts

Plasmin induces myofibroblast differentiation through glycogen synthase kinase-3β (GSK-3β) signaling [94]. In contrast, plasmin induces apoptosis of myofibroblasts [95]. Plasmin plays an important role in myofibroblast deposition in fibrosis. uPA overexpression attenuates myofibroblast differentiation in SSc fibroblasts [96], and uPA deficiency enhances myofibroblast differentiation through Endo180 and uPAR [97]. Furthermore, the induction of uPA activity in mice increases myofibroblast apoptosis [67]. In contrast, treatment with uPA increases myofibroblast differentiation [98], and the knockdown and inhibition of uPA attenuate TGF-β-induced myofibroblast differentiation [99]. uPAR deficiency in mice also increases myofibroblasts [13], and the blockade of uPAR cleavage by protease inhibitors prevents myofibroblast differentiation [100]. uPAR deficiency and silencing cause EndoMT and EMT [101,102]. In contrast, uPAR silencing by siRNA attenuates EGF-, TGF-β-, the cigarette smoke extract (CSE)-, and hypoxia-induced EMT [103,104,105,106]. The uPA/uPAR system regulates myofibroblast differentiation through multiple plasmin-dependent and plasmin-independent mechanisms.

#### 3.1.2. uPAR-Binding Protein and Myofibroblasts

uPAR can interact with several factors, including integrins, EGFR, PDGFR, VEGFR2, caveolin-1, and LRP [50].

The blockade of integrin αvβ3 by RGD peptide and the knockdown of integrin αv and β1 inhibits myofibroblast differentiation [107,108]. In addition, RGD peptide reverts EndoMT [109], and integrin α3 knockout prevents TGF-β-induced EMT [110]. In contrast, integrin α5 silencing promotes myofibroblast differentiation [111]. EGFR activation promotes an increase in myofibroblasts [112]. EGF neutralization inhibits myofibroblast proliferation [113], and the inhibition of EGFR signaling blocks EMT and EndoMT [114,115]. The inhibition of PDGFR promotes a reduction in myofibroblasts [116] and interferes with EMT [117]. In addition, neutralization of TGF-β and PDGFR signaling abolishes platelet-induced EndoMT [118]. VEGF-VEGFR2 signaling promotes EMT [119], and VEGFR2 inhibitor reverses TGF-β-induced EMT [120]. In contrast, VEGFR2 antagonism induces EndoMT [121]. The downregulation of caveolin is associated with anti-apoptotic properties of myofibroblasts [122]. Overexpression of caveolin-1 inhibits EMT [123], and the disappearance of caveolin-1 causes EMT and EndoMT [124,125]. The loss of LRP-1 promotes myofibroblast differentiation [126].

These uPAR-associated factors regulate the activation and differentiation of myofibroblasts, suggesting that the interaction with uPAR may play an important role in myofibroblast differentiation.

#### 3.1.3. Other Fibrinolytic Factors and Myofibroblasts

The uPA/uPAR system, as well as other fibrinolytic factors, such as tPA, PAI-1 and α2AP are associated with myofibroblast activation and differentiation.

tPA is a Plg activator that also converts Plg into plasmin. tPA deficiency increases apoptosis of interstitial myofibroblasts in a mouse model of obstructive injury, and tPA is associated with myofibroblast apoptosis [127]. In addition, tPA induces myofibroblast activation through LRP-1 activation [128]. 

PAI-1 regulates plasmin production by inhibiting tPA and uPA. PAI-1-specific inhibitor attenuates TGF-β-mediated myofibroblast differentiation and EMT [129]. The knockdown of PAI-1 by siRNA and PAI-1 inhibition decreases myofibroblasts [130,131]. TGF-β is known to induce PAI-1 expression and inhibit uPA, and uPA binding to PAI-1 induces myofibroblast differentiation [98]. Increases in the PAI-1 expression may promote fibrosis progression. However, it has been reported that PAI-1 deficiency increases myofibroblasts, and PAI-1 deficient ECs are more susceptible to TGF-β-induced EndoMT than wild-type ECs [132,133,134]. Elevated PAI-1 expression reportedly decreases myofibroblasts [135]. 

α2AP rapidly inactivates plasmin by inducing the formation of plasmin–α2AP complex. In contrast, α2AP deficiency attenuates dermal fibrosis in mice [80]. In addition, α2AP promotes myofibroblast differentiation and fibrosis through ATGL activation [136], and the blockade of α2AP by neutralizing antibodies or miRNA attenuates myofibroblast differentiation and fibrosis [22,137]. Furthermore, α2AP is associated with the induction of EMT and EndoMT [21,138]. α2AP may also regulate myofibroblast activation and differentiation through multiple functions.

### 3.2. Suppression of ECM Depredating Protease in Fibrosis

The suppression of ECM degradation, as well as the overproduction of ECM, causes the progression of fibrosis [10]. Proteases, including MMPs, plasmin, and uPA, regulate ECM degradation, and the activity of these proteases is inhibited by TIMPs, α2AP, and PAI-1, respectively. An imbalance between proteases and anti-proteases may promote fibrosis progression.

MMPs can be divided into six classes: collagenases, including MMP-1, MMP-8, and MMP-13; gelatinases, including MMP-2 and MMP-9; stromelysins, including MMP-3, MMP-10, and MMP-11; matrilysins, including MMP-7 and MMP-26; membrane-type MMPs, including MMP-14, MMP-15, MMP-16, MMP-17, MMP-24, and MMP-25; and others [139]. MMPs degrade ECM components, including collagen, fibronectin, laminin, entactin, tenascin, thrombospondin, and perlecan, and maintain ECM homeostasis. MMP-3 induces inactivation of α2AP and PAI-1 [19,140]. Several proteinases, including plasmin and uPA, induce the activation of MMPs. Plasmin activates MMP-1, MMP-3, MMP-9, MMP-10, and MMP-13, and uPA activates MMP-2 and MMP-9 in a plasmin-independent manner [140]. In contrast, uPAR deficiency attenuates MMP-2 and MMP-9 expression and activation [13]. Thus, the uPA/uPAR system affects MMP expression and activation.

TIMPs (TIMP-1-4) are important inhibitors of MMPs [18]. TIMPs are secreted by various cells, including immune cells, fibroblasts, and hepatocytes, and tumor necrosis factor-α (TNF-α) and prostaglandin E2 (PGE2) induce TIMP expression [10,18,141,142]. TIMP-1 and TIMP-2 levels are elevated in several types of fibrosis, including SSc, pulmonary fibrosis, liver cirrhosis, renal fibrosis, and myocardial fibrosis [14,18,65,143,144,145,146,147,148,149,150]. α2AP is elevated in fibrotic tissue, including skin and renal fibrosis [21,22,151]. Several fibrosis-associated factors, such as connective tissue growth factor (CTGF), high mobility group box 1 (HMGB1), and interferon-γ (IFN-γ) induce α2AP production [20,23,151]. The overexpression of PAI-1 attenuates ECM degradation by inhibiting the Plg activation system [152]. PAI-1 is regulated by various cytokines, such as TGF-β, EGF, and IL-1β [133]. PAI-1 expression is elevated in various types of fibrosis, including skin fibrosis, lung fibrosis, and renal fibrosis [133,153,154]. Increases in the expression of these factors may contribute to fibrosis progression by suppressing ECM degradation.

## 4. Vascular Endothelial Dysfunction in Fibrosis

Vascular endothelial dysfunction is caused by EC injury, apoptosis, defective angiogenesis and vasculogenesis, EndoMT, and excessive coagulation, leading to perivascular inflammation, tissue hypoxia, oxidative stress induction, myofibroblast accumulation, hypertension, fibrin deposition, and PARs activation [155,156,157]. 

### 4.1. The Role of the uPA/uPAR System in EC Functions 

Plasmin is known to play an important role on the maintenance of the vascular endothelial function through fibrinolysis, MMP and cytokine activation, and ECM degradation [11,158]. In addition, plasmin regulates fibrin-, MMP-, and cytokine-mediated EC proliferation, migration, and apoptosis [159,160,161]. In contrast, uPA protects ECs from apoptosis [162,163], and promotes EC proliferation [164]. The proteolytically inactive recombinant of uPA inhibits EC migration [165], and uPAR deficiency alters EC functions, including adhesion, migration, proliferation, and capillary tube formation, and decreases angiogenic functions [166]. uPAR antagonist inhibits the motility of ECs [167]. In addition, the uPA/uPAR system cross-talks with integrins and VEGFR2 and mediates EC tube formation [168]. Furthermore, uPAR-integrin interaction regulates EC migration [169], and the uPA/uPAR system is associated with growth factor-induced EC migration [170,171]. The uPA/uPAR system may regulate EC functions through plasmin-dependent or plasmin-independent mechanisms. 

### 4.2. The Role of the uPA/uPAR System in Angiogenesis

uPA regulates plasmin production and plays an important role in angiogenesis [27]. PA regulates the VEGFR1 and VEGFR2 expression by binding to haematopoietically expressed homeobox protein (HHEX) transcription factor and mediates angiogenesis [172]. uPA and uPAR shRNA enhance TIMP-1-mediated soluble VEGFR1 (sVEGFR1) secretion, and inhibit angiogenesis [173]. Plasmin regulates the vascular endothelial function through fibrinolysis, ECM degradation, and activation of growth factors [11]. In addition, plasmin can release pro-angiogenic factor VEGF from ECM and plays an important role in angiogenesis [174]. The deficiency of α2AP promotes angiogenesis through VEGF over-release in the wound-healing process [79], and α2AP causes the impairment of VEGF signaling [175]. 

Interaction of uPAR with VEGFR2 regulates VEGF signaling and promotes angiogenesis. [176]. Domain 2 of uPAR regulates uPA-mediated angiogenesis through integrin β1 and VEGFR2 [177]. uPAR also regulates factor XII-stimulated angiogenesis through integrin β1 and EGFR [178]. MMP-12 can cleave uPAR and cause the impairment of angiogenesis [179]. The interaction of uPAR with various factors may regulate its intracellular signaling and play an important role in angiogenesis. In contrast, suPAR and its ser-arg-ser-arg-tyr (SRSRY) sequence (uPAR88-92 sequence) induces angiogenesis [180]. uPAR may thus regulate angiogenesis through multiple mechanisms.

### 4.3. The Role of the uPA/uPAR System in Coagulation 

Hypercoagulation involving fibrin formation contributes to fibrosis progression [77]. The uPA/uPAR system plays a pivotal role in fibrin degradation through plasmin production. uPA and plasmin inhibitors, PAI-1 and α2AP, are elevated in several fibrotic tissue types [11], and the increase in the expression of these factors may cause the impairment of fibrinolysis through the direct inhibition of uPA and plasmin. The impairment of fibrinolysis causes fibrosis, and improvement in fibrinolysis restores fibrosis [77]. In addition, fibrin degradation product fragment can potentiate TGF-β-induced myofibroblast formation [181]. An imbalance in coagulation and fibrinolysis may thus play a critical role in fibrosis progression.

### 4.4. The Role of the uPA/uPAR System in Vascular Tone Alteration and Hypertension

Hypertension causes cardiac fibrosis [182], while fibrosis leads to pulmonary hypertension [183]. The balance between vasoconstrictor and vasodilator mediators, such as nitric oxide (NO), prostacyclin (PGI2), and endothelin-1 (ET-1), regulates vascular tone, and a shift toward vasoconstriction is associated with hypertension progression [184]. The ratio of uPA and PAI-1 is decreased in idiopathic pulmonary fibrosis patients with pulmonary hypertension [185]. uPA deficiency attenuates hypoxia-induced pulmonary arterial hypertension (PAH) progression [186]. uPAR is associated with SSc-associated PAH [187]. NO is a major vasodilation mediator, and is produced by NO synthase (NOS, including endothelial NOS [eNOS] and inducible NOS [iNOS]). uPA induces eNOS activation through LRP [188]. Treatment with UPARANT, which inhibits uPAR binding to the FPR, attenuates iNOS and NO production [189]. In contrast, iNOS and NO downregulate uPAR expression under hypoxic conditions [190]. The uPA/uPAR system may be associated with vasodilation and the onset of hypertension.

## 5. Immune Abnormalities and Inflammation in Fibrosis

Chronic inflammation leads to excessive tissue repair and triggers fibrosis progression [191]. Various stimuli, such as tissue injury, allergic response, autoimmune conditions, and infection, can cause inflammation and recruit and activate immune cells [192]. Immune cells, including T-cells, B-cells, macrophages, and dendritic cells (DCs), have been observed in fibrotic tissues [191,193]. T-cells (Th1 cells, Th2 cells, Th17 cells, and regulatory T-cells) secrete various cytokines, including IFN-γ, IL-4, IL-6, IL-12, IL-13, IL-17, and IL-22, and regulate B-cell and macrophage activation, macrophage polarization, myofibroblast differentiation, and ECM production [191,194]. B-cells induce autoantibody and cytokine production and affect EC apoptosis and ECM production [191,195]. In addition, B-cell depletion via antibodies against CD20 attenuates fibrosis progression through the suppression of M2 macrophage polarization in mice [196]. Macrophages play a pivotal role in fibrosis progression, and macrophage depletion markedly suppresses fibrosis progression [151]. Macrophages are divided into M1 and M2 macrophages subsets, and M2 macrophages are elevated under conditions of fibrotic disease [197,198]. M2 macrophage polarization is induced by IL-4 and IL-13 [199], and the inhibition of IL-4 and IL-13 signaling by IL-4Rα antibodies suppresses fibrosis progression in mice [151]. The increase in these immune cells induces production of various pro-fibrotic factors and regulates the inflammatory response, vascular homeostasis, myofibroblast differentiation, and ECM production. Abnormality of the innate and adaptive immune system is associated with fibrosis progression. 

## 6. The Role of the uPA/uPAR System in Inflammation and the Immune System

The uPA/uPAR system regulates cell recruitment, migration, and adhesion, and supports the innate and adaptive immune systems through proteolytic and non-proteolytic mechanisms [200]. Plasmin induced by the uPA/uPAR system has both pro- and anti-inflammatory effects and regulates chemotaxis, invasion, phagocytosis, and cytokine production through PAR-1 or Annexin A2 in various cell types, including monocytes, macrophages, and DCs [201,202,203]. In addition, Plg/plasmin regulates macrophage activation, polarization and efferocytosis [204,205]. Furthermore, plasmin can activate complement factors (C3 and C5), factor XII, and MMPs [203]. Plasmin is also associated with the polarization of T cells [206], and mediates the innate and adaptive immune systems.

uPA regulates macrophage chemotaxis, neutrophil activation, and migration through uPAR-dependent or uPAR-independent mechanisms [207]. uPA also mediates the inflammatory response, such as inflammatory cytokine production and suppression of the NF-κB pathway through plasmin activation [34,200]. Furthermore, uPA induces the production of M2 phenotype macrophages [208]. In contrast, uPAR interacts with several receptors, including integrins and LRP, and participates in the initiation of the innate immune response through the induction of cell adhesion and migration [200]. In addition, uPAR regulates toll-like receptor (TLR) 2, 4, and 7 signaling, and affects inflammation (cytokine production, mediation of NF-κB pathway) and immune responses (neutrophil and macrophage activation, macrophage efferocytosis) [38,209,210,211]. The expression and release of suPAR is induced by inflammation and immune activation [212]. The uPAR-derived ser-arg-ser-arg-tyr (SRSRY) peptide (uPAR88-92 sequence) can interact with FPR1 and is associated with chemokine regulation and monocyte migration [212,213]. The uPA/uPAR system also affects T-cell priming and T-cell effector function [200]. 

This system has multiple functions and contributes to the inflammatory response and innate and adaptive immune responses. 

## 7. Conclusions and Therapeutic Perspectives

The uPA/uPAR system regulates proteolysis and intracellular signal transduction through multiple plasmin-dependent and plasmin-independent mechanisms, and mediates vascular homeostasis, the immune system, and ECM homeostasis. This review has presented the latest findings, showing that the uPA/uPAR system plays an important role in fibrosis progression. uPA and uPAR are reportedly associated with several pro-fibrotic events, including vascular endothelial dysfunction (EC injury, apoptosis, defective angiogenesis, EndoMT, and excessive coagulation), immune abnormalities (excessive immune activation, immune cells infiltration, and autoantibodies production), and myofibroblast differentiation. In addition, the importance of uPA and uPAR in fibrosis progression has been proven in several animal models. The control of uPA and uPAR expression, uPA/uPAR binding, and uPAR cleavage may improve vascular endothelial dysfunction, immune abnormalities, myofibroblast and ECM deposition, and fibrosis. The regulation of the uPA/uPAR system through several methods, such as neutralizing antibodies, miRNA, peptides, and protease inhibitors, may be a novel therapeutic approach to managing fibrotic diseases. Further investigations will be required to clarify the role of the uPA/uPAR system in fibrotic diseases.

## Data Availability

Not applicable.

## Abbreviations

α2AP	α2-antiplasmin
AMPK	AMP-activated protein kinase
ATF	amino-terminal fragment
CKD	chronic kidney disease
CSE	cigarette smoke extract
CTGF	connective tissue growth factor
DC	dendritic cell
ECM	extracellular matrix
EGF	epidermal growth factor
EGFR	epidermal growth factor receptor
EMT	epithelial-to-mesenchymal transition
EndoMT	endothelial-to-mesenchymal transition
eNOS	endothelial NOS
ET-1	endothelin-1
ERK	extracellular-signal-regulated kinase
FRP	formyl peptide receptor
FSGS	focal segmental glomerulosclerosis
GSK-3β	glycogen synthase kinase-3β
HGF	hepatocyte growth factor
HMGB1	high mobility group box 1
IGF1R	insulin-like growth factor 1 receptor
IFN	interferon
iNOS	inducible NOS
JAK	janus kinase
JNK	c-Jun N-terminal kinase
LRP	low-density lipoprotein receptor
MMT	macrophage-to-myofibroblast transition
MMP	matrix metalloproteinase
MSC	mesenchymal stem cells
NO	nitric oxide
NOS	NO synthase
PAH	pulmonary arterial hypertension
PAI-1	plasminogen activator inhibitor-1
PAR	protease-activated receptor
PDGF	platelet-derived growth factor
PDGFR	platelet-derived growth factor receptors
PI3K	phosphatidylinositol 3-kinase
Plg	plasminogen
PGE2	prostaglandin E2
PGI2	prostacyclin
SLE	systemic lupus erythematosus
SSc	systemic sclerosis
STAT	signal transducer and activator of transcription protein
suPAR	soluble uPAR
TGF-β	transforming growth factor-β
TIMPs	tissue inhibitors of MMPs
TNF-α	tumor necrosis factor-α
uPA	urokinase plasminogen activator
uPAR	urokinase plasminogen activator receptor
VEGF	vascular endothelial growth factor
VEGFR	vascular endothelial growth factor receptor
